# Supplementary figures and images for: The Drosophila Nab2 RNA binding protein inhibits m6A methylation and male-specific splicing of Sex lethal transcript in female neuronal tissue
Source: eLife. 2023 Jul 17;12:e64904. doi: 10.7554/eLife.64904 (PMC10351920; doi:10.7554/eLife.64904)

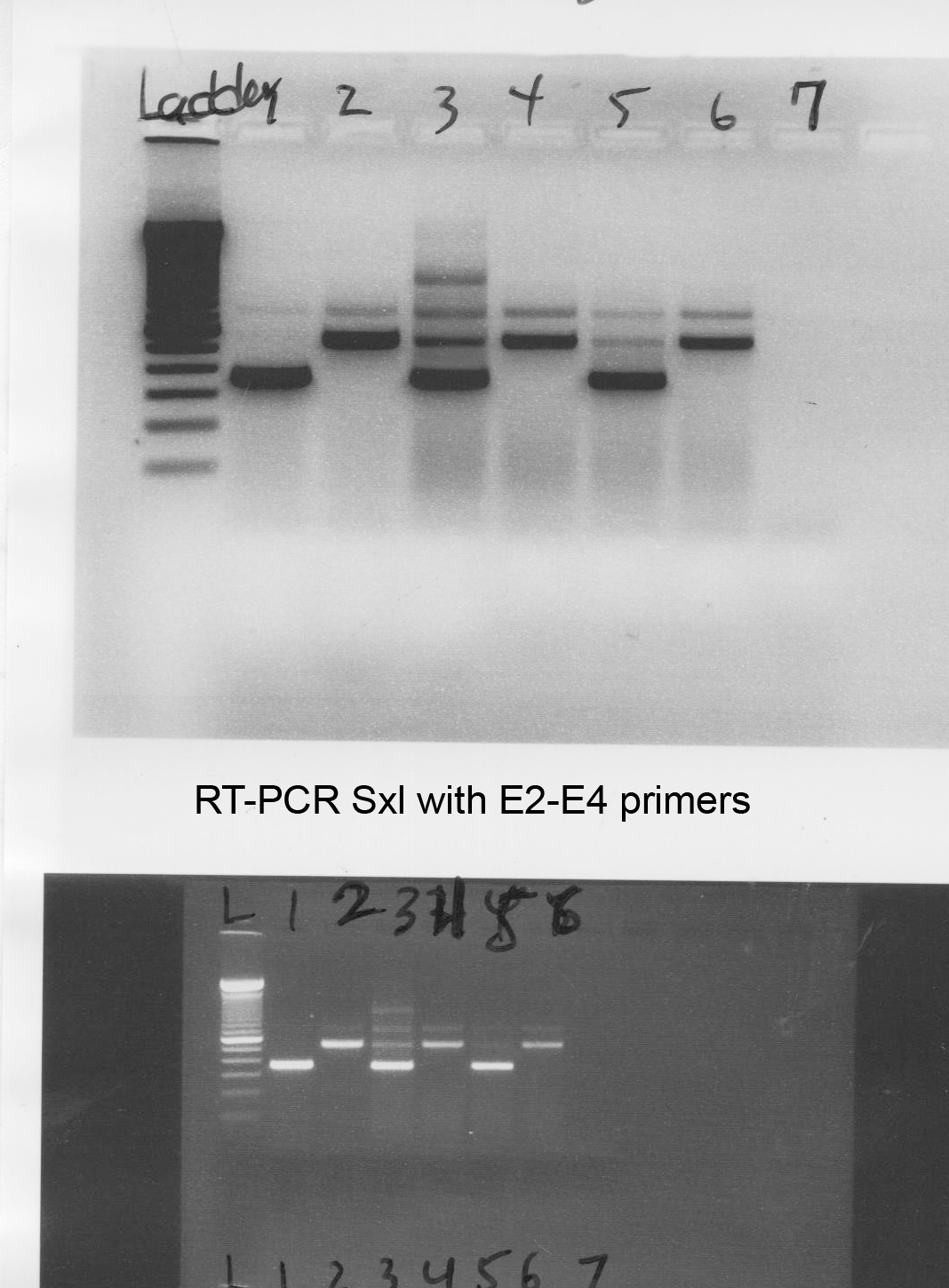

Supplement: Figure 3—source data 1. — Shown as original (bottom) and inverted (top) grayscale. [file elife-64904-fig3-data1.zip › Fig 3B source RT-PCR Sxl.tif]

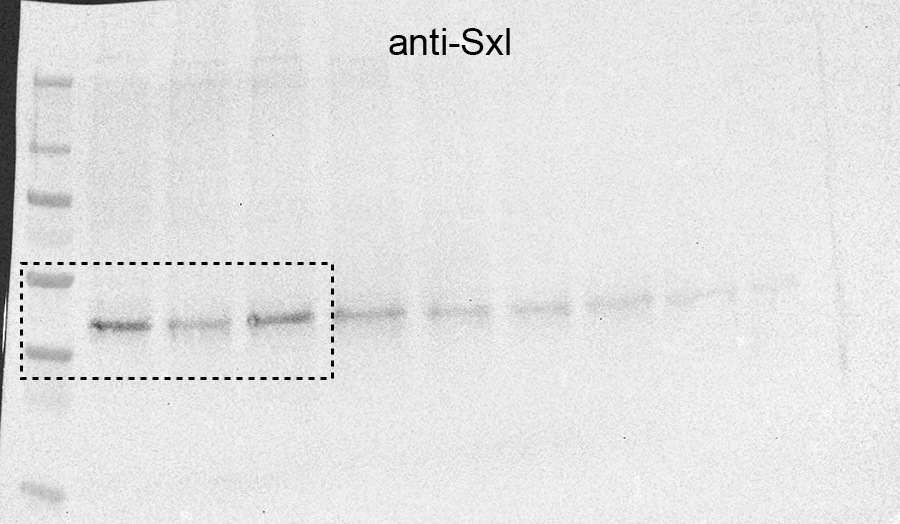

Supplement: Figure 3—source data 2. — Boxed areas correspond to regions shown in the main figure. [file elife-64904-fig3-data2.zip › Fig 3D source anti-Sxl blot #1.tif]

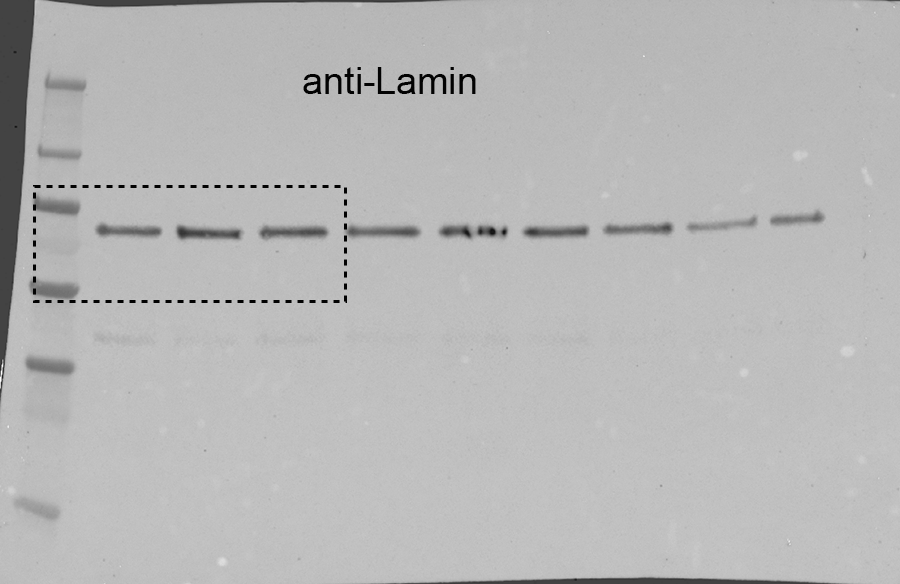

Supplement: Figure 3—source data 3. — Boxed areas correspond to regions shown in the main figure. [file elife-64904-fig3-data3.zip › Fig 3D source anti-lamin blot #1.tif]

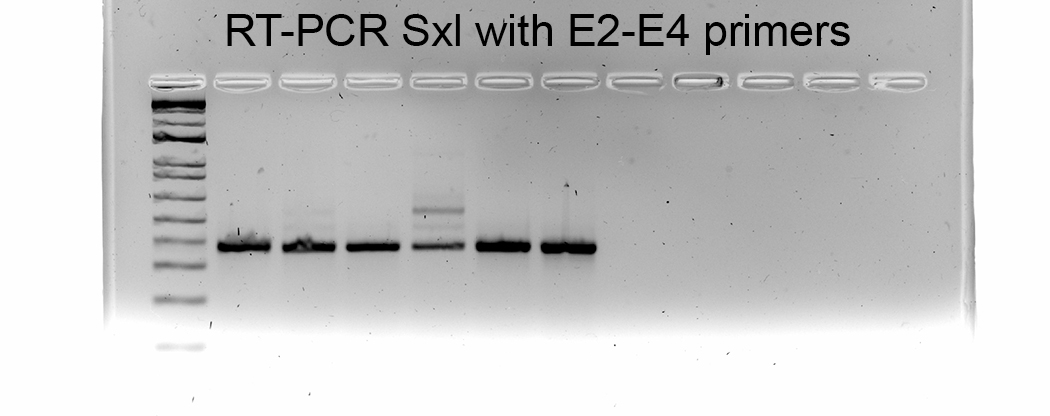

Supplement: Figure 3—source data 4. [file elife-64904-fig3-data4.zip › Fig 3F source RT-PCR Sxl.tif]

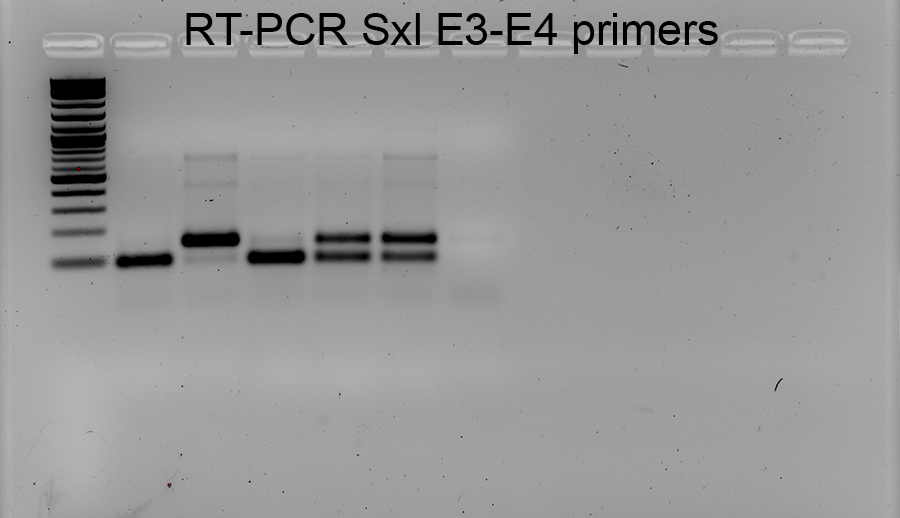

Supplement: Figure 6—source data 1. [file elife-64904-fig6-data1.zip › Fig 6H source RT-PCR Sxl.tif]

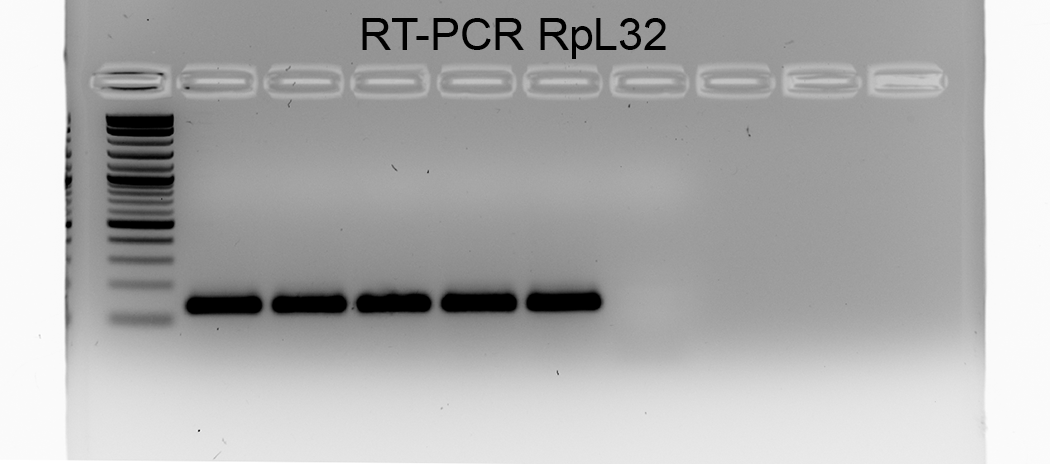

Supplement: Figure 6—source data 2. [file elife-64904-fig6-data2.zip › Fig 6H source RT-PCR RpL32.tif]

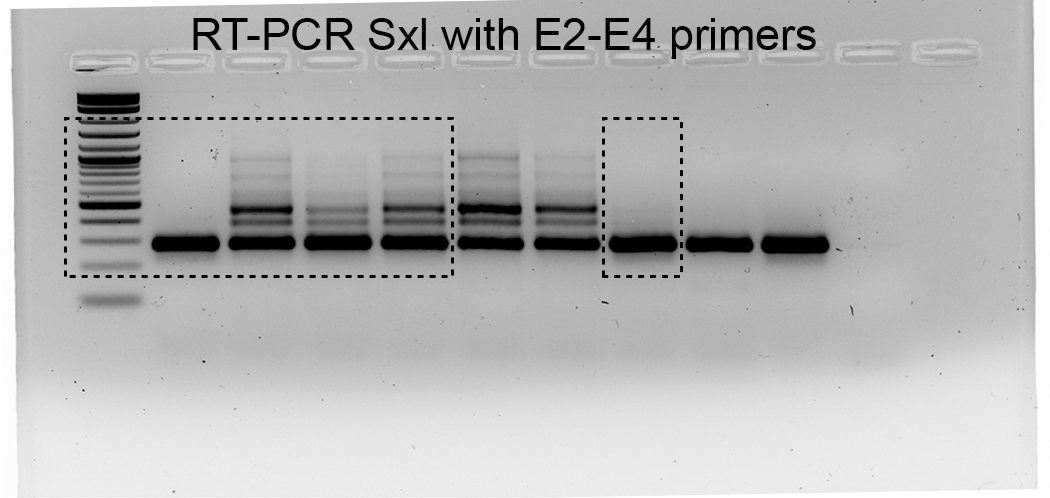

Supplement: Figure 6—figure supplement 3—source data 1. — Boxes correspond to regions included in the figure supplement. [file elife-64904-fig6-figsupp3-data1.zip › Fig S9b source RT-PCR Sxl.tif]

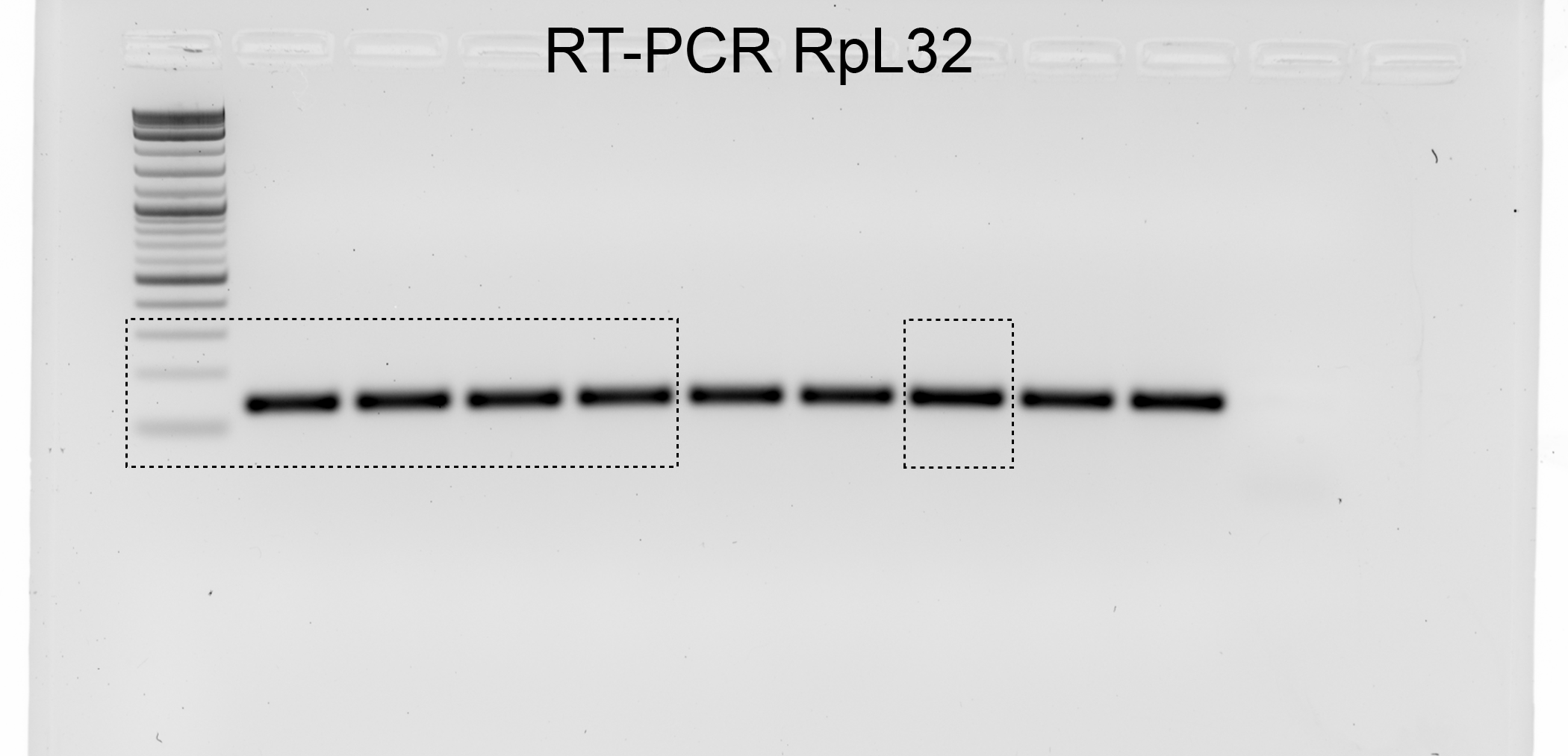

Supplement: Figure 6—figure supplement 3—source data 2. — Boxes correspond to regions included in the figure supplement. [file elife-64904-fig6-figsupp3-data2.zip › Fig S9B source RT-PCR RpL32.tif]
